# Melatonin enhances drought tolerance in lemongrass: physiological, biochemical, and antioxidant mechanisms

**DOI:** 10.1038/s41598-026-41870-z

**Published:** 2026-04-28

**Authors:** Mohammad Javad Nazarideljou, Kamyar Lahijani, Seyran Farabi, Shahin Hamzehzadeh, Hiva Aspoukeh

**Affiliations:** Institute of Agriculture, Water, Food, and Nutraceuticals, Mah.C., Islamic Azad University, Mahabad, Iran

**Keywords:** Growth regulator, Environmental stress, Medicinal plants, Antioxidants, Oxidative markers, Biochemistry, Physiology, Plant sciences

## Abstract

Drought stress is a critical constraint on plant growth and productivity in arid and semi-arid regions, significantly impairing the physiological traits and antioxidant capacity of medicinal plants. Lemongrass (*Cymbopogon citratus*), a medicinal and aromatic plant valued for its antioxidant and antimicrobial properties, is highly sensitive to environmental stresses. Melatonin, a multifunctional molecule involved in growth regulation and stress tolerance, shows promise in mitigating drought-induced damage; however, its application in medicinal plants remains underexplored. This study investigated the effects of foliar-applied melatonin (0, 100, and 200 µM) on lemongrass under varying drought stress levels (100%, 70%, and 40% field capacity) in controlled greenhouse conditions. Physiological parameters (chlorophyll content, stomatal conductance, relative water content), growth indices (leaf and root dry weight), compatible solutes (proline), oxidative stress markers (electrolyte leakage, hydrogen peroxide, malondialdehyde), and antioxidant responses (catalase, peroxidase, total phenols, total antioxidant capacity) were assessed. Results revealed that drought stress significantly reduced chlorophyll content (by up to 56.6%), stomatal conductance (by 42.6%), and plant biomass. Conversely, foliar application of melatonin, particularly at 200 µM, effectively mitigated these adverse effects, increasing biomass by 9–11%, improving leaf water status by 14–15%, enhancing proline accumulation, and reducing oxidative stress markers. Furthermore, melatonin enhanced catalase and peroxidase activities as well as phenolic accumulation in a concentration-dependent manner. These findings indicate that melatonin, by enhancing antioxidant defense systems and maintaining physiological homeostasis, offers a promising strategy for improving drought tolerance and supporting the sustainable production of medicinal plants under water-limited conditions.

## Introduction

Climate change-driven alterations in precipitation patterns, rising temperatures, and intensified anthropogenic pressures have markedly increased the frequency and severity of abiotic stresses affecting global agriculture. Among these stresses, drought represents one of the most pervasive and damaging constraints, particularly in arid and semi-arid regions where water availability is already limited^1,2^. The progression to acute drought stress is often a cascade initiated by these climatic changes, where reduced rainfall and higher evapotranspiration directly deplete soil moisture, a condition frequently exacerbated by interacting abiotic stresses such as heat and salinity. Although drought, salinity, temperature extremes, and metal toxicity share common physiological and biochemical stress pathways^3,4^, most notably oxidative stress induction and disruption of cellular homeostasis, drought uniquely combines hydraulic failure with metabolic and oxidative impairments, making it especially detrimental to crop productivity and sustainability^5,6^. Consequently, drought stress has been consistently associated with substantial reductions in plant growth, biomass accumulation, yield stability, and phytochemical quality across a wide range of horticultural and medicinal species^7,8^.

At the physiological and biochemical levels, drought stress disrupts plant performance through impaired water uptake, reduced stomatal conductance, degradation of chlorophyll pigments, and limitation of photosynthetic efficiency, ultimately constraining carbon assimilation and growth^9^. These effects are frequently accompanied by membrane destabilization, electrolyte leakage, and severe oxidative stress caused by excessive accumulation of reactive oxygen species (ROS), such as hydrogen peroxide (H_2_O_2_)^10,11^. Elevated ROS levels promote lipid peroxidation, reflected by increased malondialdehyde (MDA) accumulation, and damage proteins, pigments, and nucleic acids unless efficiently detoxified by antioxidant defense systems^5^. Plants counteract these effects by activating enzymatic antioxidants, including catalase (CAT) and peroxidase (POD), alongside non-enzymatic antioxidants and osmoprotectants such as proline and phenolic compounds, which collectively contribute to cellular protection and stress tolerance^12,13^.

To mitigate drought-induced damage, diverse management strategies have been explored, including genetic improvement, optimized irrigation practices, microbial inoculation, and the exogenous application of plant growth regulators and biostimulants^6,14^. Among these approaches, melatonin (N-acetyl-5-methoxytryptamine) has recently gained considerable attention due to its multifunctional role in plant stress physiology. Melatonin is an endogenous indoleamine that acts as both a powerful free-radical scavenger and a central regulator of antioxidant defense networks, modulating ROS homeostasis under abiotic stress conditions^15^. Exogenous melatonin application has been shown to enhance drought tolerance by maintaining stomatal conductance, preserving chlorophyll pigments, improving relative water content, stabilizing cellular membranes, and stimulating antioxidant enzyme activities, thereby sustaining physiological integrity under water deficit^16,17^. Unlike many synthetic protectants, melatonin is effective at low concentrations, environmentally safe, and capable of coordinating both enzymatic (CAT, POD) and non-enzymatic antioxidant responses, as well as osmotic adjustment through increased proline accumulation^18,19^. Recent comprehensive reviews further highlight melatonin’s integrative role in drought mitigation through regulation of redox balance, membrane integrity, and stress-responsive signaling pathways^20,21^.

Lemongrass (*Cymbopogon citratus* (DC.) Stapf), a perennial aromatic medicinal plant of the Poaceae family, is extensively cultivated for its essential oils rich in citral and geraniol, compounds with well-documented antimicrobial, anti-inflammatory, and antioxidant properties^22^. Despite its adaptation to tropical and subtropical climates, lemongrass is highly sensitive to water scarcity, with drought stress severely limiting vegetative growth, biomass production, and essential oil yield and quality^23,24^. Although melatonin-mediated drought tolerance has been widely investigated in major agronomic and horticultural crops, no study to date has evaluated its physiological, biochemical, and antioxidant roles in lemongrass, representing a clear and important research gap in medicinal plant science.

Therefore, this study is the first to systematically investigate the effects of foliar-applied melatonin on drought-stressed lemongrass by integrating morpho-physiological, biochemical, and antioxidative analyses. We hypothesized that melatonin enhances drought tolerance in lemongrass by improving water status and stomatal regulation, reducing membrane damage and oxidative stress, and activating antioxidant defense systems. Specifically, the objectives of this study were to: (i) evaluate the effects of melatonin on growth-related and physiological traits, including leaf and root dry weight, stomatal conductance, chlorophyll pigments, relative water content, and ion leakage; (ii) quantify drought-induced biochemical and oxidative stress markers, including proline, H_2_O_2_, and MDA; and (iii) characterize melatonin-induced modulation of antioxidant defenses through CAT, POD, total phenolic content, and DPPH radical scavenging activity.

## Results

### Physiological parameters, compatible solutes, and yield

Photosynthetic pigments and stomatal conductance were significantly influenced by drought stress and melatonin application, although their interaction was not statistically significant. The highest chlorophyll content and stomatal conductance were observed at 100% field capacity (FC) with 200 µM melatonin, differing significantly from other treatments. Moderate (70% FC) and severe (40% FC) drought stress reduced total chlorophyll content by 36.3% and 56.6%, respectively, compared to the control (100% FC). Similarly, stomatal conductance decreased by 30.6% and 42.6% under moderate and severe drought, respectively (Table 1).

**Table 1:** Effects of drought stress (field capacity) and foliar melatonin application on chlorophyll pigments and stomatal conductance in lemongrass.

Treatments		Chlorophyll pigments (mg/g FW)			Stomatal conductance (mM/m^2^/s)
		a	b	Total	
Field capacity (%)	100	1.19 ± 0.09a*	1.37 ± 0.10a	2.56 ± 0.19a	50.37 ± 1.43a
	70	0.75 ± 0.04b	0.87 ± 0.04b	1.63 ± 0.08b	42.63 ± 0.94b
	40	0.52 ± 0.02c	0.60 ± 0.02c	1.11 ± 0.04c	30.16 ± 1.62c
Melatonin (µM)	0	0.69 ± 0.08c	0.80 ± 0.09c	1.49 ± 0.17c	37.14 ± 3.09c
	100	0.81 ± 0.10b	0.94 ± 0.11b	1.75 ± 0.21b	40.71 ± 3.04b
	200	0.96 ± 0.14a	1.11 ± 0.16a	2.07 ± 0.30a	45.31 ± 2.93a

*Mean values within each column sharing the same letter are not significantly different according to Duncan’s multiple range test at the 5% probability level.

**Fig. 1:**
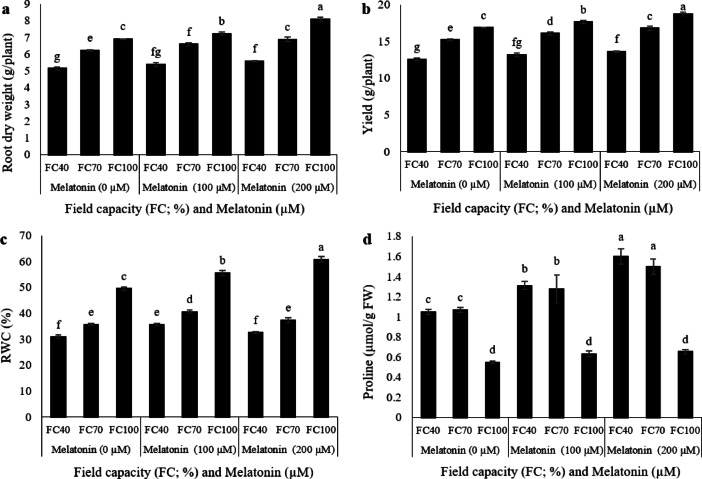
Effects of drought stress (as a percentage of field capacity, FC) and foliar melatonin application on (**a**) root dry weight, (**b**) leaf dry weight (yield), (**c**) relative water content (RWC) and (**d**) proline content of lemongrass. Values are means ± SE (n = 3).

Drought stress, melatonin treatment, and their interaction significantly affected shoot and root dry weights. The highest root dry weight was recorded at 100% FC with 200 µM melatonin. Raising melatonin from 100 to 200 µM produced a stronger positive effect (Fig. 1a). A similar trend was observed for shoot dry weight, representing lemongrass yield. Increasing drought severity reduced biomass, whereas foliar melatonin, particularly at 200 µM, consistently improved yield and alleviated drought-induced losses (Fig. 1b). Melatonin-induced biomass increases under well-watered (100% FC), moderate (70% FC), and severe (40% FC) conditions were approximately 11%, 10%, and 9%, respectively, compared to untreated plants at the same moisture level.

Relative leaf water content (RWC), an indicator of plant water status, was significantly influenced by the treatments. The highest RWC was observed at 100% FC, and the lowest under severe drought. Foliar melatonin application partially restored plant water status under stress. Specifically, 100 µM melatonin increased RWC by 14% and 15% under moderate and severe drought, respectively, compared to untreated controls (Fig. 1c). Proline, a key osmoregulatory metabolite, was significantly affected by the interaction of drought stress and melatonin. The highest proline content was recorded under severe drought (40% FC) with 200 µM melatonin, and the lowest under well-watered conditions melatonin application (Fig. 1d).

### Oxidative stress and membrane damage

Electrolyte leakage, hydrogen peroxide (H_2_O_2_), and malondialdehyde (MDA) contents, indicators of oxidative stress and lipid peroxidation, were significantly affected by the interaction of drought stress and melatonin. As drought severity increased, these markers rose significantly. However, 100 µM melatonin reduced electrolyte leakage by 13.5% and 24% under moderate and severe drought, respectively, compared to untreated plants. Similarly, 200 µM melatonin more effectively lowered H_2_O_2_, decreasing accumulation by 17% and 12% under moderate and severe drought, respectively, compared to untreated controls Table 2.

**Table 2:** Mitigating role of melatonin on oxidative stress markers and membrane damage indicators in lemongrass under drought stress.

Melatonin (µM)	Field capacity (%)	Ion leakage (%)	Hydrogen peroxide (%)	Malondialdehyde (µM/g FW)
0	40	33.66 ± 0.38a*	65.99 ± 0.35a	21.16 ± 0.10a
	70	20.77 ± 0.31d	30.63 ± 1.07d	14.66 ± 0.45d
	100	9.45 ± 0.13f	20.50 ± 0.35g	7.23 ± 0.21g
100	40	29.14 ± 0.35c	60.37 ± 0.33b	19.63 ± 0.22b
	70	15.82 ± 0.10e	27.77 ± 0.62e	12.01 ± 0.08e
	100	8.94 ± 0.58f	22.19 ± 0.49g	6.07 ± 0.28h
200	40	31.01 ± 0.39b	58.12 ± 0.98c	16.65 ± 0.68c
	70	15.54 ± 0.74e	25.46 ± 0.41f	10.28 ± 0.46f
	100	9.41 ± 0.46f	21.25 ± 0.22g	6.19 ± 0.20gh

*Mean values within each column sharing the same letter are not significantly different according to Duncan’s multiple range test at the 5% probability level.

MDA levels doubled and tripled under moderate and severe drought, respectively, compared to the control. However, 200 µM melatonin markedly decreased MDA levels, reducing accumulation by 30% and 71% under moderate and severe stress, respectively, compared to untreated controls.

### Antioxidant defense responses

**Fig. 2:**
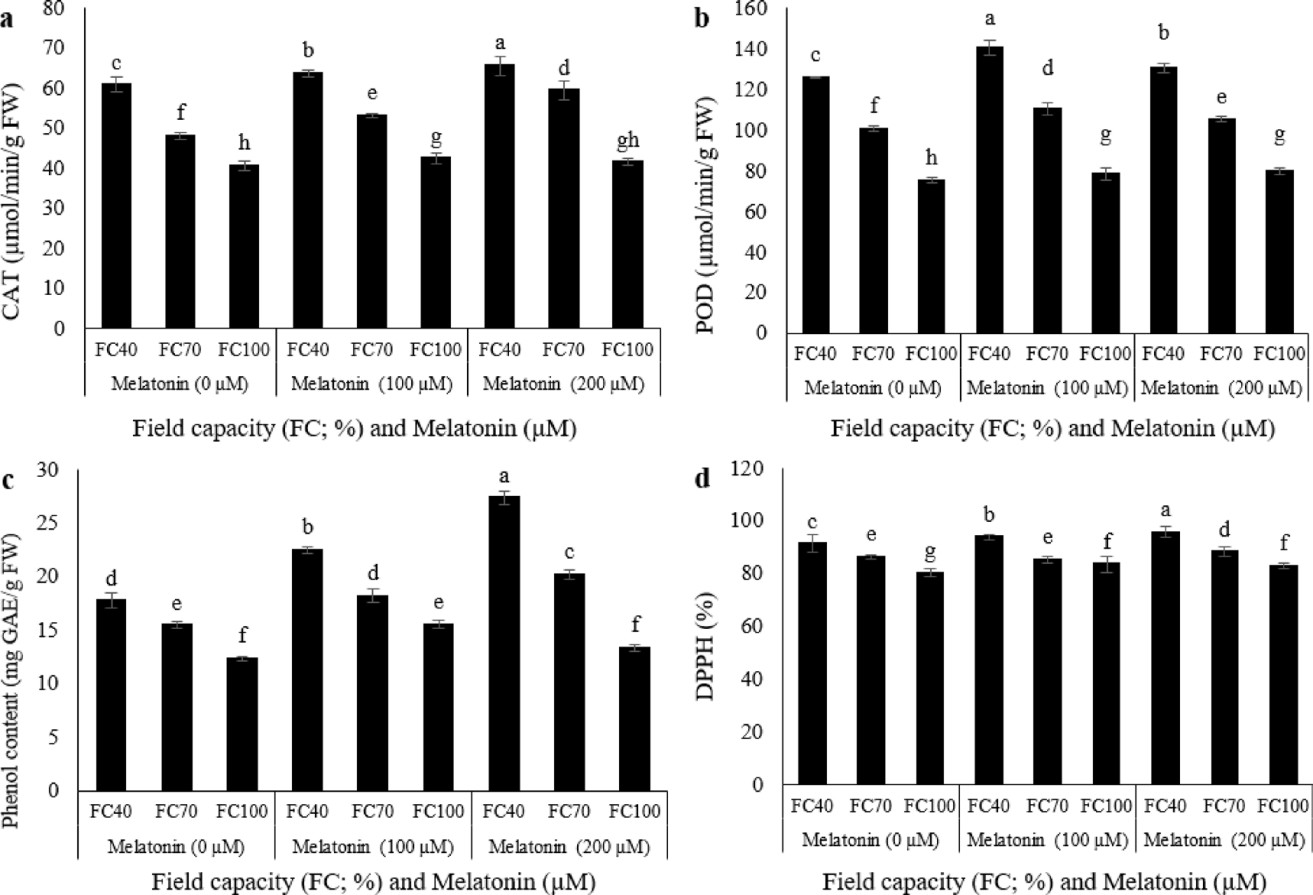
Effects of melatonin on antioxidant defense indicators in lemongrass under drought stress (as a percentage of field capacity, FC): (**a**) catalase activity, (**b**) peroxidase activity, (**c**) total phenolic content, and (**d**) antioxidant capacity (DPPH).

Enzymatic and non-enzymatic antioxidant defenses in lemongrass were significantly enhanced by drought stress and further strengthened by melatonin. The highest catalase (CAT) activity was observed under severe drought with 200 µM melatonin (Fig. 2a), while the highest peroxidase (POD) activity occurred with 100 µM melatonin under severe drought (Fig. 2b). Total phenolic content, a non-enzymatic antioxidant, increased with drought and melatonin treatments. The highest Total phenolic content was recorded under severe drought (40% FC) with 200 µM melatonin (Fig. 2c). Antioxidant capacity, assessed via the DPPH assay, increased with drought severity and melatonin concentration. The highest antioxidant capacity (95.4%) was observed under severe drought with 200 µM melatonin, and the lowest (80.2%) under well-watered conditions without melatonin (Fig. 2d).

## Discussion

### Physiological traits and biomass

Our findings demonstrate that drought stress significantly disrupted physiological functioning, growth, and antioxidant defenses in lemongrass (*Cymbopogon citratus*), while foliar melatonin application, particularly at 200 µM, substantially mitigated these effects. The examination of physiological, biochemical, and antioxidant responses provides critical insights into melatonin’s mechanisms for enhancing drought tolerance in lemongrass.

Drought stress significantly reduced total chlorophyll content, stomatal conductance (gₛ), and relative water content (RWC), whereas melatonin, especially at 200 µM, effectively improved these parameters. Chlorophyll degradation under drought likely results from chloroplast structural damage, enhanced chlorophyllase activity, impaired biosynthesis^25^, and reactive oxygen species (ROS) accumulation^26^. Reduced stomatal conductance under drought reflects an adaptive response to minimize transpirational water loss^27^, but limits photosynthesis and growth. Similarly, decreased RWC in drought-stressed plants indicates impaired water uptake and cellular water retention, reducing turgor pressure and stomatal function^28^. Melatonin treatment enhanced chlorophyll content, stomatal conductance, and plant water status, likely due to its roles in strengthening antioxidant defenses, protecting photosynthetic pigments, optimizing stomatal function, and promoting root development for improved water uptake. These findings align with previous studies showing melatonin’s capacity to protect chloroplasts from oxidative damage by scavenging ROS, thereby maintaining photosynthetic efficiency^29^. Naghizadeh et al.^30^ reported similar melatonin-mediated protection against chlorophyll degradation in drought-stressed *Dracocephalum moldavica*. Moreover, melatonin’s regulation of osmolytes (e.g., proline) and cellular water retention likely optimized stomatal function and gas exchange under water deficit^19^. These results position melatonin as an effective regulator of plant stress resilience. While this study provides strong evidence for melatonin’s role in mitigating drought stress by measuring key physiological pathways, including stomatal conductance and photosynthetic pigments as indicators of photosynthetic capacity, a limitation lies in the absence of direct measurements of key gaseous exchange parameters such as the net photosynthetic assimilation rate and transpiration. Future research should address these measurements to offer a more comprehensive mechanistic understanding.

Drought-induced reductions in leaf and root dry weights correlated with diminished photosynthetic capacity and impaired water status. These findings are consistent with Li et al.^31^, who linked drought-induced declines in stomatal conductance and chlorophyll content to reduced photosynthesis and productivity. Melatonin counteracted these effects by preserving chlorophyll content and stomatal function, thereby sustaining photosynthetic performance. Furthermore, melatonin-induced proline accumulation and enhanced antioxidant defenses likely contributed to improved biomass production, supporting its roles in stabilizing physiological processes, stimulating growth, alleviating oxidative damage, and maintaining osmotic homeostasis^16,31,49^.

### Oxidative stress and antioxidant defenses

Electrolyte leakage serves as a reliable indicator of plasma membrane integrity, with increased leakage reflecting structural damage to cellular membranes. Drought stress reduces cellular relative water content and elevates ROS levels, which disrupt membrane phospholipids, increase fluidity, and compromise membrane stability and permeability^9,26^. Hydrogen peroxide (H_2_O_2_), a primary ROS, is produced at low levels under normal conditions and rapidly scavenged by antioxidant systems. However, drought-induced reductions in water potential and disruptions in photosynthetic light reactions markedly increase H_2_O_2_ production^32^. Unneutralized H_2_O_2_ can cause severe oxidative damage to cellular structures^33^. MDA, a byproduct of lipid peroxidation in polyunsaturated fatty acids, is a widely recognized biomarker of oxidative membrane damage^34^. In this study, exogenous melatonin application at 200 µM significantly reduced electrolyte leakage, H_2_O_2_, and MDA levels in drought-stressed lemongrass. As a potent antioxidant and multifunctional plant regulator, melatonin mitigates oxidative stress by directly scavenging ROS and upregulating antioxidant enzymes, including CAT and POD, thereby enhancing plant defense against oxidative damage^17,35,36^. These mechanisms collectively suppressed ROS accumulation, prevented lipid peroxidation, and reduced MDA content. Furthermore, the observed protective effect of melatonin on membrane integrity likely results from its ability to prevent the oxidative degradation of unsaturated fatty acids and stabilize membrane structure under drought conditions^37^.

Drought-induced oxidative stress in lemongrass was also characterized by increased ROS production, particularly H_2_O_2_, resulting from reduced water potential and restricted gas exchange. This led to cellular oxidative stress, which damaged biomembranes and organelles. In response, lemongrass activated key enzymatic antioxidant defenses including CAT and POD, to detoxify excess H_2_O_2_ and other ROS and maintain cellular integrity^32^. Importantly, H_2_O_2_ also functions as a signaling molecule, mediating the activation of stress-responsive genes through specific signaling cascades. Additionally, increased total phenolic content under drought conditions enhanced the plant’s non-enzymatic antioxidant system, contributing to the scavenging of free radicals, inhibition of lipid peroxidation, and maintenance of redox homeostasis^38^. Thus, upregulation of antioxidant enzyme activity and phenolic compound biosynthesis represents a coordinated and adaptive response to minimize oxidative damage and maintain physiological functionality during drought stress^39^.

In addition, our findings demonstrate that exogenous melatonin significantly enhances the antioxidant defense capacity of lemongrass under drought stress. Melatonin upregulates antioxidant enzymes (CAT and POD), presumably through transcriptional enhancement of antioxidant gene expression and activation of drought-related signaling pathways^17,31^. Additionally, melatonin promotes enzyme stability by increasing compatible solute accumulation, such as proline, which prevents enzymatic deactivation under oxidative stress. Melatonin also stimulates phenylalanine ammonia-lyase (PAL) activity, activating the phenylpropanoid pathway and enhancing phenolic compound biosynthesis. These phenolics scavenge ROS, inhibit lipid peroxidation, and stabilize the cellular redox environment^40^. Thus, melatonin exerts both direct ROS-scavenging activity and indirect regulatory effects on the antioxidant defense network, orchestrating a multilayered and synergistic protective response that mitigates oxidative injury, maintains membrane integrity, and enhances drought resilience.

Interestingly, in the present study, CAT activity peaked at 200 μM melatonin, whereas POD activity was highest at 100 μM. This variation likely reflects each enzyme’s differential responsiveness to melatonin concentrations and its dose-dependent regulatory effects. Such responses may be influenced by differences in gene regulation, protein stability, or redox feedback mechanisms at distinct concentrations of melatonin. Antioxidant enzyme responses to melatonin vary with enzyme type, plant species, stress severity, and physiological context^16,17^.

## Conclusion

In summary, melatonin functions as an effective biostimulant for mitigating drought stress in lemongrass (*Cymbopogon citratus*) by preserving photosynthetic pigments, improving plant water status, limiting membrane damage, and enhancing antioxidant defenses. These integrated physiological and biochemical responses highlight melatonin’s potential as a valuable agrochemical tool for managing abiotic stress in medicinal plant cultivation. Given its consistent efficacy, particularly at 200 µM, melatonin could be incorporated into crop management programs to maintain biomass production and safeguard essential oil quality under water-limited conditions, thereby offering growers a practical and low-toxicity strategy to enhance stress resilience.

Future research should focus on identifying optimal application timing and frequency for field-scale production, examining long-term impacts on soil–plant interactions, and determining melatonin’s effects on both the yield and compositional profile of lemongrass essential oil. In addition, comprehensive transcriptomic and metabolomic analyses are needed to elucidate the underlying regulatory pathways and to refine melatonin-based management practices across diverse medicinal and aromatic plant species.

## Materials and methods

### Plant material, growth conditions, and treatments

Lemongrass (*Cymbopogon citratus* (DC.) Stapf) transplants were obtained from the Zargiah Medicinal Plant Center, Shiraz, Fars Province, Iran. Transplants were transferred into polyethylene pots (20 cm diameter) filled with standard agricultural soil (Table 3). Plants were maintained under optimal irrigation for two months to ensure establishment and rooting. Subsequently, drought stress was imposed at three soil moisture levels: 100%, 70%, and 40% of field capacity (FC).

Foliar applications of melatonin (0, 100, and 200 µM) were initiated concurrently with drought treatments and applied biweekly for three months. Each experimental unit comprised six plants, with treatments arranged in three replicates (18 plants per treatment). Greenhouse conditions were controlled: day/night temperatures of 26/19 ± 2 °C, relative humidity of 65 ± 5%, and natural light.

**Table 3:** Physicochemical properties of the soil used in the experiment.

Physical properties				Chemical properties						
Soil texture (%)				Saturation percent	Organic carbon (%)	pH	EC (dS/m)	N (%)	P (ppm)	K (ppm)
Clay	Silt	Sand	Texture							
11	31	58	Loam	48	3.1	7.3	0.77	0.23	25.5	286

### Growth and morphological measurements

Five months after initiating the experiment, plants were randomly sampled for physiological, morphological, and biochemical analyses. Samples were immediately frozen in liquid nitrogen and stored at − 80 °C until analysis. At harvest, shoots (leaves) and roots were separated, and morphological traits, including leaf and root dry weights, were measured. Photosynthetic capacity was assessed by measuring chlorophyll content and stomatal conductance in fully expanded mature leaves. Chlorophyll a, b, and total chlorophyll were quantified following Dere et al.^41^. Stomatal conductance and gas exchange were measured using a porometer (LP2402, Decagon, USA). Relative water content (RWC) was determined according to Ali Dib et al.^42^, based on fresh, turgid, and dry weights.

### Oxidative damage assessment

Oxidative damage was evaluated using electrolyte leakage (EL), hydrogen peroxide (H_2_O_2_), and malondialdehyde (MDA) content. Electrolyte leakage was measured following Lutts et al.^43^. Leaf discs were rinsed with distilled water, incubated in 10 mL of distilled water for 2 h, and initial conductivity (EC₁) was recorded. Samples were then autoclaved at 120 °C for 30 min to measure final conductivity (EC_2_). Electrolyte leakage (%) was calculated as:

EL (%) = (EC_1_/EC_2_) × 100.

Hydrogen peroxide content was determined following Velikova et al.^44^. Leaf tissue (0.1 g) was homogenized in 2 mL of 0.1% (w/v) trichloroacetic acid (TCA), centrifuged at 12,000 × g for 15 min, and the supernatant was mixed with phosphate buffer (pH 7.0) and potassium iodide (KI). Absorbance was measured at 390 nm, and H_2_O_2_ content was calculated using a standard curve.

Lipid peroxidation was assessed by measuring MDA content using the thiobarbituric acid (TBA) method^45^. Leaf tissue (0.2 g) was homogenized in 2 mL of 0.1% TCA, centrifuged, and the supernatant was mixed with TBA reagent (0.5% TBA in 20% TCA). The mixture was incubated at 95 °C for 30 min, cooled, and absorbance was recorded at 532 and 600 nm. MDA concentration was calculated using an extinction coefficient of 155 mM⁻^1^ cm⁻^1^.

### Biochemical and antioxidant analyses

Proline content, an indicator of osmotic stress, was quantified following Bates et al.^46^. Leaf tissue (50 mg) was homogenized in 2 mL of 70% ethanol, heated at 100 °C for 10 min, and centrifuged at 4000 × g for 20 min. The supernatant was mixed with ninhydrin reagent, and absorbance was measured at 520 nm. Proline concentration was determined using a standard curve.

Total phenolic content was measured using the Folin–Ciocalteu method^47^. Methanolic extract (5 µL) was mixed with 1200 µL of 10% Folin–Ciocalteu reagent, 960 µL of 7% sodium carbonate, and 180 µL of distilled water. Samples were incubated for 30 min at room temperature in the dark, and absorbance was read at 760 nm. Total phenolic content was expressed as mg gallic acid equivalents (GAE) per gram fresh weight.

Antioxidant capacity was evaluated using the DPPH (2,2-diphenyl-1-picrylhydrazyl) radical scavenging assay^48^. Methanolic extract (400 µL) was added to 2 mL of 0.1 mM DPPH in 80% methanol, incubated for 30 min at 25 °C in the dark, and absorbance was measured at 517 nm. Radical scavenging activity was calculated as:

$$ {\text{DPPH Scavenging Activity }}\left( \% \right) = \left[ {\left( {{\mathrm{AC}} - {\mathrm{AS}}} \right)/{\mathrm{AC}}} \right] \times {1}00. $$

where AC is the absorbance of the control (DPPH without extract), and AS is the absorbance of the sample.

### Antioxidant enzyme assays

Antioxidant enzymes were extracted from fully expanded mature leaves. Fresh leaf tissue (0.5 g) was homogenized in an ice bath using 5 mL of ice-cold 50 mM sodium phosphate buffer (pH 7.8) supplemented with 1 mM EDTA and 2% (w/v) polyvinylpyrrolidone (PVP). The homogenate was centrifuged at 15,000 × g for 15 min at 4 °C, and the resulting supernatant was used as the crude enzyme extract.

Catalase (CAT) activity was assayed following the method of Aebi (1984) by measuring the decline in absorbance associated with H_2_O_2_ decomposition at 240 nm for 1 min using a UV–visible spectrophotometer. The 3 mL reaction mixture consisted of 2.9 mL of 50 mM phosphate buffer (pH 7.0), 0.1 mL of 30 mM H_2_O_2_, and 50 µL of enzyme extract. The reaction was initiated by adding the enzyme extract. One unit of CAT activity was defined as the amount of enzyme required to decompose 1 µmol of H_2_O_2_ per minute under the assay conditions.

Peroxidase (POD) activity was determined according to Chance and Maehly (1955) by recording the increase in absorbance due to guaiacol oxidation at 470 nm. The reaction mixture contained 1 mL of 50 mM phosphate buffer (pH 6.5), 200 µL of 1% guaiacol, 100 µL of 0.3% H_2_O_2_, and 50 µL of enzyme extract, yielding a final volume of 1.35 mL. The reaction was initiated by adding H_2_O_2_, and the change in absorbance at 470 nm was monitored for 1 min. One unit of POD activity was defined as an absorbance increase of 0.1 per minute.

### Statistical analysis

Data were analyzed using SAS software (version 9.2; SAS Institute Inc., Cary, NC, USA). The experiment followed a factorial arrangement in a completely randomized design with three replications. Factors included drought stress levels and melatonin concentrations. Means were compared using Duncan’s multiple range test at *p* ≤ 0.05 and *p* ≤ 0.01. 

## Data Availability

The data that support the findings of this study are available from the corresponding author on reasonable request.
